# Assessing the Link between Maternal Transport Modes and Childhood Mortality in Nigeria

**DOI:** 10.1007/s10995-024-03963-x

**Published:** 2024-06-15

**Authors:** Oluwaseun Addie, Kehinde F Seun-Addie, Samuel Ojima Adejoh, Adetayo Olorunlana

**Affiliations:** 1 Department of Geography, University of Ibadan, Ibadan, Nigeria; 2 National Population Commission of Nigeria, Headquarters, Abuja, Nigeria; 3 Department of Social Work, University of Lagos, Lagos, Nigeria; 4 Department of Social Work, University of Free State, Bloemfontein, South Africa; 5 Caleb University, Imota, Lagos Nigeria

**Keywords:** Child mortality, Maternal transport, Sustainable development goals, Nigeria

## Abstract

**Objectives:**

The study explored the association between maternal transport modes and childhood mortalities in Nigeria.

**Method:**

Utilizing data and definitions from the 2018 Nigeria Demographic and Health Survey report, the ten-year early mortality rates of the five childhood mortalities and the percentage of live births in the 5 years before the survey, transported by eight identified means of transportation, were statistically correlated for each of Nigeria’s 36 states and the federal capital territory (FCT) in the *R* environment at a significance level of α < 0.05.

**Results:**

In the spatial distribution of the five childhood mortalities, a notable north-south dichotomy was observed, contrasting with the spatial spread of maternal transport modes. The five childhood mortalities exhibited a significant, moderately positive correlation with transportation by Private Car or Truck, while their associations with Public Transport or Bus and Walking were notably moderate but negative.

**Conclusion for Practice:**

While the use of private cars or trucks should be encouraged as a means of maternal transport, public transport should be better organized to provide efficient services to women who need such services for maternal and child healthcare. Additionally, steps should be taken to reduce travel distances to health facilities to manageable distances for mothers.

## Significance

**What is Already Known on this Subject?:**

Maternal- and child mortality remain global health concerns, with sub-Saharan Africa and South Asia bearing the brunt. Transportation has been identified as a significant barrier to healthcare accessibility for women and children in the region, especially in rural areas.

**What this Study adds?:**

Maternal transport mode is significantly associated with childhood mortalities. Efficient public transport has the potential of reducing child mortality; likewise locating healthcare facilities in a way to make them trekking distances for mothers.

## Introduction

Goal 3 of the Sustainable Development Goals (SDG) is to ensure healthy lives and promote well-being for all ages (United Nations, 2015). Its first and second targets are the reduction of maternal mortality and ending all preventable deaths under five years of age. However, sub-Saharan Africa and South Asia account for more than 80% of maternal deaths worldwide (UNICEF, 2021a).

Central to achieving the first and second targets of SDG Goal 3 in sub-Saharan Africa is improved access to healthcare (Rajé, 2018; Dotse-Gborgbortsi et al., 2020; Wariri et al., 2021). There is a growing consensus that transport is a significant barrier to healthcare accessibility for women and children in the region, especially in rural areas (Babinard & Roberts, 2006; Coleman et al., 2010; Fiagbe et al., 2012; Bhopal et al., 2013; Godefay et al., 2016; Ehiri et al., 2018; Sacks et al., 2021).

While some studies have examined the effect of transport on maternal mortality (Atuoye et al., 2015; DeSisto et al., 2021), and others have investigated the link between transport mode and child mortality (Tette et al., 2020; Quattrochi et al., 2020), the association of the mode of maternal transport and child mortality is sparsely examined.

Studies have established an association between distances from health facilities and child and maternal mortality (Atuoye et al., 2015; Karra et al., 2017; Quattrochi et al., 2020), with distances beyond a threshold resulting in a significant number of deaths (Bhusal et al., 2011; Samson, 2012; Lerberg et al., 2014; Karra et al., 2017). To address the challenge of long distances from health facilities and the excessive time spent by women and children trying to reach them, there is a need for efficient and comfortable transport (Godefay et al., 2016; Sacks et al., 2021).

Highlighting the role of rapid transportation in reducing maternal mortality, Peters et al. (2018) reported that in a cohort of 4105 pregnant women using a novel maternal transportation arrangement in rural Sierra Leone and Liberia, all women with maternal complications survived while 96% of newborns lived. In contrast, Fiagbe et al. (2012) found that in a Ghanaian municipality, 65.0% of pregnant women used public transport to reach the healthcare facility for delivery, 28.9% walked, 3.7% used personal cars, and 1.6% arrived on motorbikes.

Although Ekpenyong, Matheson, & Serrant (2022) examined transportation modes among women with obstetric complications in Nigeria’s South-South Zone and reported that 91.2% used motorized transport while 8.8% walked to healthcare facilities, they noted that the categorization of transport modes was overly generalized, obscuring variations in motorized transport types.

Tette et al. (2020) examined, among other things, the mode of transporting neonates admitted to district and regional hospitals in Ghana. They found that 41.7% of the neonates who died were transported by public buses, while 36.0% of the survivors arrived by taxis. The study concluded that the chosen mode of transportation may have compromised the health of the deceased infants. This conclusion is supported by Weddih et al.‘s (2019) study in Mauritania, where a significant portion of the infants who died (34.7%) were brought to healthcare facilities after birth, potentially compromising their health due to the mode of transport.

Koffi et al. (2017) reported that 34.2% of the 2,057 child deaths recorded in the 2013 Nigerian DHS were attributed to the unavailability of transport. In a broader context, Karra et al. (2017) studied the relationship between facility distance and child mortality in 21 low- and middle-income countries. They suggested that prompt transportation positively impacts child mortality by reducing the time taken to transport sick children to appropriate healthcare facilities.

Although Karra et al. (2017) collected data on the most common mode of transportation used by people in villages or communities to reach healthcare facilities, the study did not focus on transport modes. Instead, this information was utilized to determine distance and travel time to healthcare facilities.Thus, this study aimed to use data on maternal transport modes and childhood mortalities from the 2018 Nigerian DHS report to determine the association, or otherwise, of maternal transport modes and cases of childhood mortalities across the states in the country.

## Methods

### Study Area

With a population of over 200 million, Nigeria is the most populous country in Africa and is projected to become the third most populous country in the world, following China and India, by the year 2050 (World Population Review, 2022). The country is divided into 36 states and a federal capital territory (FCT), which are grouped into six geo-political zones (NPC & ICF, 2019). The country operates a decentralized healthcare system comprising public and private health providers. The prime goal is to strengthen primary healthcare, the first tier of its three-tier healthcare system. The national healthcare policy is focused on delivering qualitative, effective, efficient, equitable, accessible, affordable, acceptable, and comprehensive healthcare to the people (NPC & CIRCLE, 2020).

### Conceptual Definitions

The 2018 NDHS report categorized childhood deaths into five, with appropriate definitions. Neonatal mortality is the probability of a newborn dying within the first month of life. In contrast, infant mortality is the probability of a newborn dying between birth and the first birthday. Post-neonatal mortality is the probability of a newborn dying between the first month of life and the first birthday, and it is computed as the difference between infant and neonatal mortality. Child mortality is the probability of a newborn dying between the first and the fifth birthday, while under-5 mortality is the probability of a newborn dying between birth and the fifth birthday (NPC & ICF, 2019; p. 164).

### Data Collection

The 2018 NDHS report (NPC & ICF, 2019), aimed to provide current estimates of basic demographic and health indicators to assist policymakers in evaluating and planning health programs and policies. The survey utilized a stratified sample selected in two stages, with 74 sampling strata created by dividing the 36 states and the Federal Capital Territory into urban and rural areas. Data extracted from the report included ten-year early childhood mortality rates, covering neonatal, post-neonatal, infant, child, and under-5 mortality rates per 1,000 live births. Additionally, information on means of transportation to health facilities for delivery was extracted from the maternal health care section, detailing the percentage of live births delivered in health facilities in the 5 years before the survey and the modes of transportation used, such as private cars or trucks (P.T), taxis or paid drivers or tricycles (T.P.T), motorcycles or scooters (M.S), public transport or bus (PT.B), bicycles (B), boats or canoes (B.C), walking (W) and ambulance or other modes of transportations (A.O), across all 36 states of Nigeria and the FCT.

### Analysis

In *R*, the mortality and transport mode data for the states and the FCT were mapped to reveal the spatial pattern of each phenomenon across the country using the *sf* package (Pebesma, 2018), *dplyr* package (Hadley, Romain, Lionel, and Kirill, 2021), and *tmap* package (Tennekes, 2018). The *sf* package was utilized to import shapefiles containing the boundaries of the 36 states and the FCT. Subsequently, the mutating joins function in *dplyr* was employed to link the mortality and transport mode data to each state and the FCT. The thematic map of each mortality variable was then generated using various functions in *tmap*.

Pairing each of the eight identified means of transportation in turn with the five categories of childhood mortality resulted in 40 pairs, for which tests for association between the paired data were conducted. A correlation analysis was implemented with the aid of the *cor.test* function in R. The method was set to “pearson,” utilizing Pearson’s product-moment correlation coefficient *cor(x, y)* and following a *t*-distribution with length *(x)-2* degrees of freedom. An asymptotic confidence interval based on Fisher’s Z transform (R Core Team, 2021) was provided with α set to 0.05. The coefficient of correlation (*r*) describes the strength and direction of an association between variables; however, it is important to note that correlation does not imply causality (Schober et al., 2018). Correlation ranges from + 1 to -1, with signs indicating whether the correlation is positive or negative; values closer to both extremes signify a stronger correlation. The convention for interpreting correlation coefficients is presented in Table 1.

**Table 1:** Adopted convention for interpreting correlation coefficient

Correlation Coefficient	Interpretation
0 < |*r*| < 0.3	weak correlation
0.3 ≤ |*r*| < 0.7	moderate correlation
|*r*| ≥ 0.7	strong correlation

This manuscript is based on secondary data that was not based on clinical study or patient data, there was no need to obtain ethical approval.

## Results

Of the five childhood mortality categories, the infant group had the highest rates, followed by the child mortality and neonatal groups. The least observed rates were recorded in the post-neonatal group. Concerning women who experienced neonatal, post-neonatal, infant, child, and under-5 mortality, the rates were highest among those aged 40–49 years, followed by those under 20 years of age. Next were those aged 30–39 years old, while those aged 20–29 years had the lowest rates. Women with six children or more witnessed the highest mortality rates in all categories, followed by those having a child for the first time. Women with 4–5 children were next in mortality rates, while those with 1–2 children had the lowest mortality rates. Additional details can be found in Table 2.

**Table 2:** Family and social characteristics of the childhood mortality

Mortality	NN	PNN	Infant	Child	Under 5
*Mother’s age at birth*					
< 20	53	32	85	82	160
20–29	32	26	59	65	120
30–39	34	29	64	65	124
40–49	55	38	94	81	168
*Birth order*					
1	47	23	69	50	116
2–3	30	24	54	59	110
4–6	33	30	64	69	129
7+	47	42	89	111	190
*Mother’s education*					
No Education	40	36	77	101	170
Primary	38	28	66	56	118
Secondary	32	19	51	27	77
> Secondary	28	16	44	12	56
*Wealth quintile*					
Lowest	39	39	78	103	173
Second	41	36	77	99	169
Middle	41	27	69	64	128
Fourth	32	21	53	35	86
Highest	28	12	40	14	53
*Zone*					
N-Central	32	26	58	39	95
N-East	37	37	73	65	134
N-West	46	35	80	117	187
S-East	27	22	48	29	75
S-South	27	21	49	25	73
S-West	31	12	43	20	62

N-Central = North-central, N-East = North-east, N-West = North-west, S-East = South-east, S-South = South-south, S-West = South-west, NN = Neo-natal, PNN = Post-neonatal

**Fig. 1:**
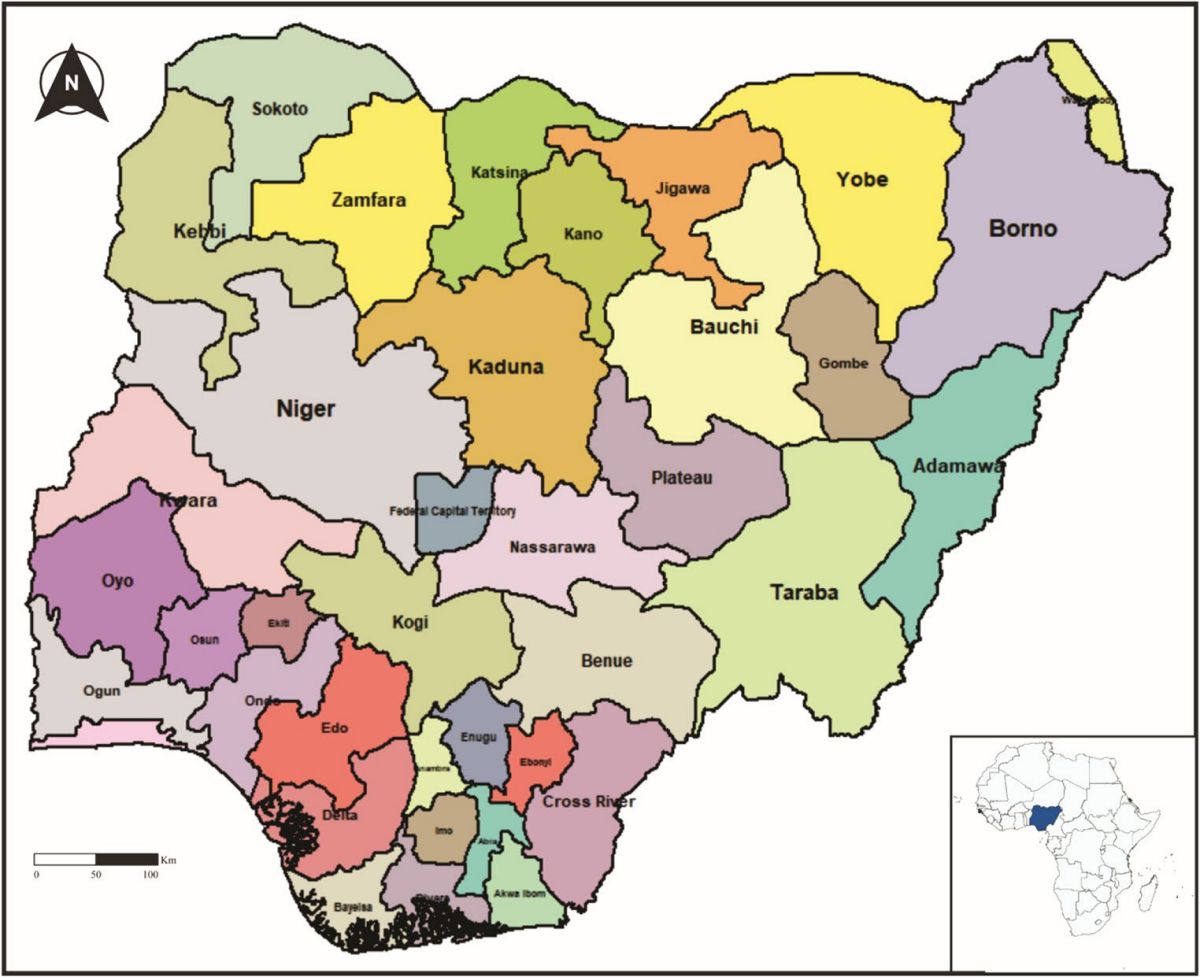
Map of Nigeria, showing the 36 States and the Federal Capital Territory

**Fig. 2:**
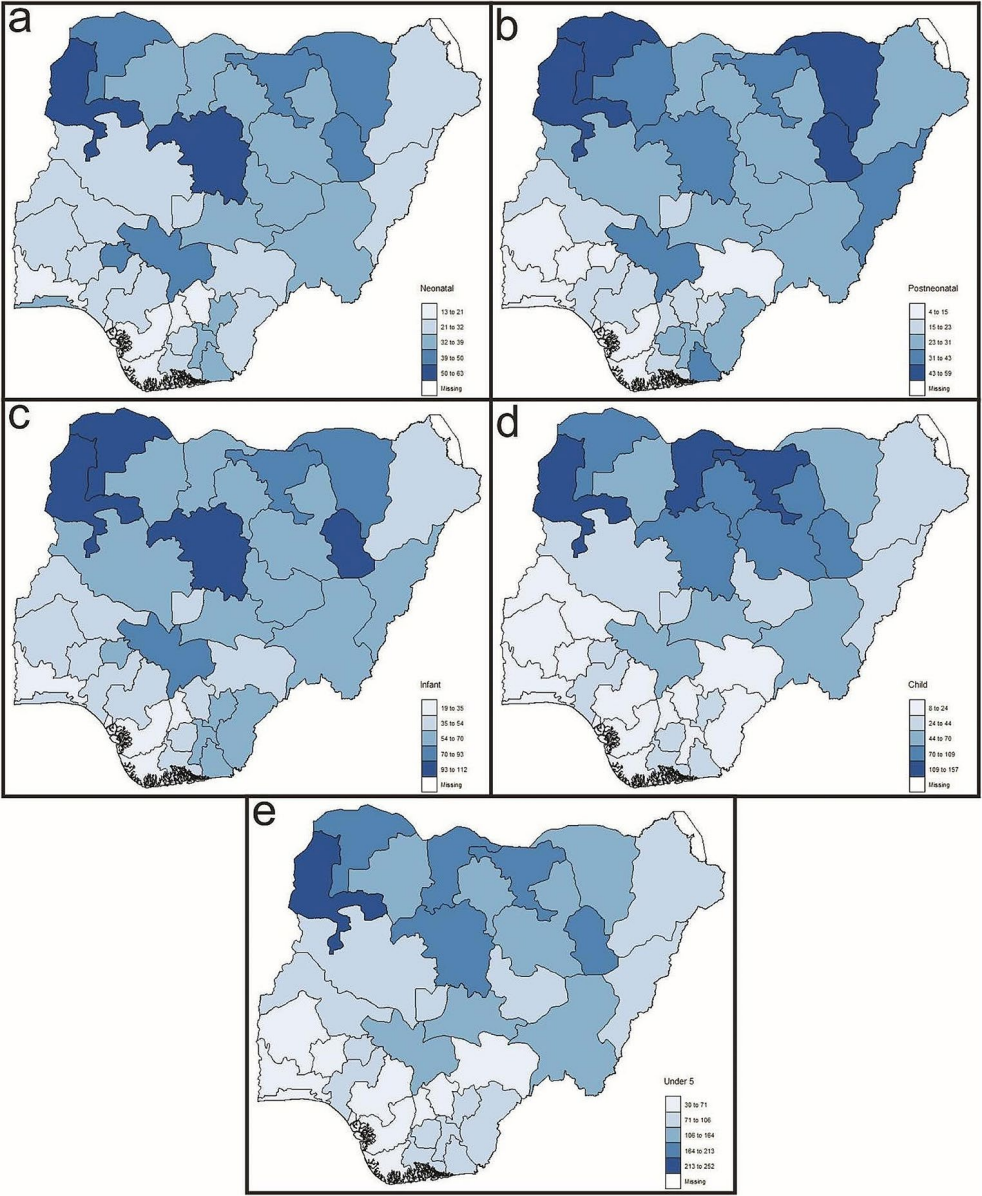
Spatial distributions of (**a**) neonatal, (**b**) post-neonatal, (**c**) infant, (**d**) child, and (**e**) under-5 mortalities in Nigeria

The states (Fig. 1) with high neonatal mortality rates (Fig. 2a) in the country were concentrated in the northern part. Concerning post-neonatal mortality (Fig. 2b), the highest rates were also observed in the north of Nigeria. Infant mortality (Fig. 2c), akin to the previous mortality categories, had its highest rates concentrated in states in the northern part of the country. Similarly, states in the north exhibited the highest child mortality rates (Fig. 2d). The north-south dichotomy was observable in Under-5 mortality (Fig. 2e) as well.

**Fig. 3:**
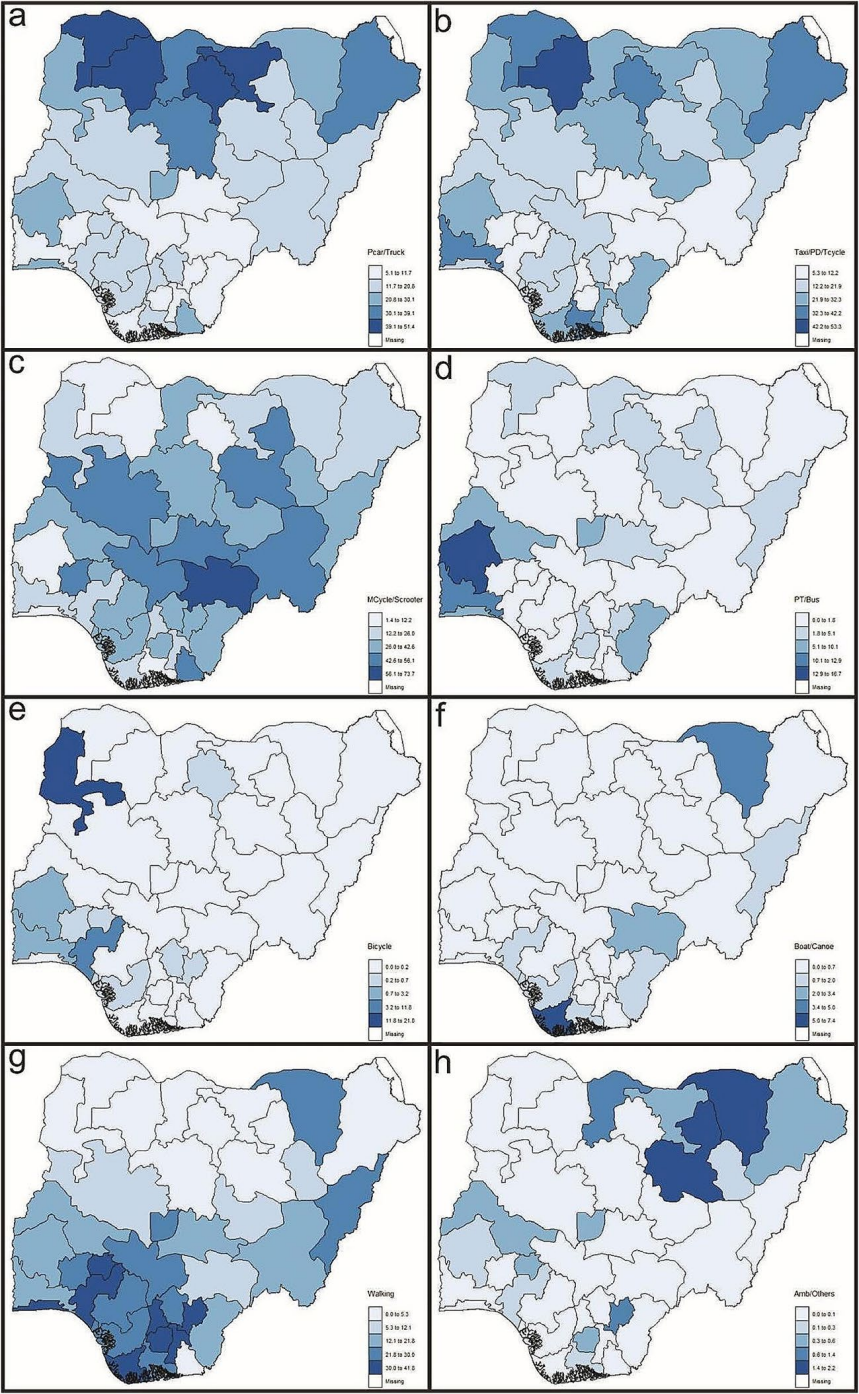
Spatial distributions of transportation to delivery facilities by: (**a**) private car or truck, (**b**) taxi or paid drivers or tricycle), (**c**) motorcycle or scooter, (**c**) public transport or bus, (**d**) bicycle, (**e**) boat or canoe, (**f**) walking and (**g**) ambulance or other

With regard to the means of transporting pregnant women to the health facility where delivery occurred, the highest percentages of women transported by P.T (Fig. 3a) were concentrated in the north, while the states with the lowest percentages were in the south. Most women transported by T.PD.T (Fig. 3b) were residents of states in the north, with a few outliers outside the region. Mothers transported by M.S (Fig. 3c) to deliver their newborns were mainly concentrated in the middle and southern parts of the country. PT.B (Fig. 3d) was more commonly used in the southwestern part of the country to transport women to health facilities for delivery. Among the eight identified means of transporting pregnant women for delivery, the Bicycle (Fig. 3e) was the least utilized. In 19 states and the FCT, its use was not recorded, and in the remaining 17 states where it was used, 76.5% recorded less than 1% usage, with 64.7% in the southern region.

Using a B.C (Fig. 3f) to transport pregnant women was noted in 15 states across the country, with a usage percentage of about 1% in 53.3% of them. Women who walked (Fig. 3g) to health facilities for delivery were predominantly from states in the southern part of the country, with Zamfara being the only state where walking was not recorded as a means of transportation for pregnant women. The use of A.O (Fig. 3h) to take expecting mothers for delivery was observed in 16 states and the FCT, with higher percentages mainly concentrated in the North-eastern part of the country. However, in 76.5% of these states, the percentage usage of A.O was about 1%.

The result of the correlation analysis showed that there were statistically significant positive associations between transportation by P.T. and the five childhood mortality categories. Concerning transportation by T.P.T and each of the five childhood mortality categories, the associations, though not statistically significant, were weak and positive, except for Child mortality. In contrast to the two previous modes of transportation, transportation by M.S to the place of delivery was negatively associated with each of the five childhood mortality categories but not statistically significant. Transportation by PT.B had a statistically significant negative association with each identified childhood mortality. Transportation by B and each of the five childhood mortality categories had positive associations, but not statistically significant. The associations were negative but not statistically significant for maternal transportation by B.C and each identified childhood mortality category. Walking to the healthcare facility of delivery and each of the five childhood mortality had statistically significant negative associations. In contrast to the last two modes of transportation, maternal transportation by A.O was positively associated with all the childhood mortality categories, though not statistically significant. For more details, refer to Table 3.

**Table 3:** Result of correlation of childhood mortality and means of transportation to health facilities where delivery took place

Variables	*r*	*ρ*	95% CI	Variables	*r*	*ρ*	95% CI
P.T, Child	0.66	< 0.0001	[0.419, 0.807]	B, Child	0.32	0.0553	[-0.001, 0.582]
P.T, Infant	0.44	0.0061	[0.138, 0.670]	B, Infant	0.24	0.1590	[-0.095, 0.520]
P.T, NN	0.44	0.0065	[0.135, 0.668]	B, NN	0.21	0.2114	[-0.122, 0.500]
P.T, PNN	0.40	0.0142	[0.087, 0.641]	B, PNN	0.24	0.1549	[-0.092, 0.522]
P.T, Under 5	0.61	< 0.0001	[0.357, 0.780]	B, Under 5	0.30	0.0746	[-0.030, 0.566]
T.P.T, Child	0.32	0.0521	[-0.002, 0.585]	B.C, Child	− 0.24	0.1469	[-0.526, 0.088]
T.P.T, Infant	0.21	0.2216	[-0.123, 0.497]	B.C, Infant	− 0.25	0.1429	[-0.528, 0.085]
T.P.T, NN	0.13	0.4611	[-0.207, 0.431]	B.C, NN	− 0.32	0.0537	[-0.583, 0.005]
T.P.T, PNN	0.25	0.1425	[-0.085, 0.528]	B.C, PNN	− 0.15	0.3819	[-0.450, 0.185]
T.P.T, Under 5	0.29	0.0801	[-0.036, 0.562]	B.C, Under 5	− 0.26	0.1188	[-0.539, 0.069]
M.S, Child	− 0.18	0.2923	[-0.474, 0.155]	W, Child	− 0.65	< 0.0001	[-0.807, -0.419]
M.S, Infant	− 0.03	0.8726	[-0.348, 0.299]	W, Infant	− 0.49	0.0022	[-0.701, -0.195]
M.S, NN	− 0.01	0.9645	[-0.331, 0.317]	W, NN	− 0.44	0.0059	[-0.671, -0.140]
M.S, PNN	− 0.05	0.7828	[-0.365, 0.281]	W, PNN	− 0.47	0.0037	[-0.686, -0.166]
M.S, Under 5	− 0.12	0.4636	[-0.431, 0.208]	W, Under 5	− 0.63	< 0.0001	[-0.791, -0.383]
PT.B, Child	− 0.36	0.0286	[-0.612, -0.041]	A.O, Child	0.21	0.2181	[-0.125, 0.498]
PT.B, Infant	− 0.43	0.0085	[-0.659, -0.119]	A.O, Infant	0.13	0.4421	[-0.202, 0.436]
PT.B, NN	− 0.35	0.0359	[-0.602, -0.025]	A.O, NN	0.13	0.4323	[-0.120, 0.438]
PT.B, PNN	− 0.44	0.0059	[-0.672, -0.140]	A.O, PNN	0.12	0.4882	[-0.215, 0.425]
PT.B, Under 5	− 0.41	0.0120	[-0.647, -0.097]	A.O, Under 5	0.20	0.2486	[-0.138, 0.488]

NN = Neonatal, PNN = Post-Neonatal, P.T = Private Car or Truck, T.P.T = Taxi or Paid Driver or Tricycle, M.S = Motorcycle or Scooter, PT.B = Public Transport or Bus, B = Bicycle, B.C = Boat or Canoe, W = Walking, A.O = Ambulance or Others

## Discussion

This study examined the relationship between maternal transportation modes and childhood mortality in Nigeria using data from the 2018 NDHS. The results revealed a north-south divide in childhood mortality cases nationwide. While the northern region had high rates of all five childhood mortalities, the southern states exhibited lower rates. Interestingly, despite this trend, the northern part of the country had a higher percentage of usage of seemingly superior maternal transportation modes (P.T, T.P.T, and A.O), while the south relied more on less comfortable modes (M.S, PT.B, B, B.C, and W), but with better outcomes.

The statistically significant moderate positive association between transportation by P.T and each of the five childhood mortalities suggests that the higher the use of a private car or truck, the higher the childhood mortalities, even though the use of P.T gives the individual control of time. However, our finding is at variance with previous studies (Okwaraji & Edmond, 2012; Ehiri et al., 2018; Peters et al., 2018; Sacks et al., 2021). Two probable reasons may be adduced for the finding: the socio-economic status of those using P.T and the distance from the delivery places. Most women that used P.T were from rich households, i.e., from the fourth and the highest wealth quintiles (Table 4), suggesting that those from poor households, which were likely to be more in number, were left out. Also, since childhood mortalities and the use of P.T were higher in the north (Fig. 3a), it might be safe to assume that the places of delivery in the region were far from the majority of pregnant women, necessitating the use of automobiles with the attendant high cost of transportation (Ehiri et al., 2018), an assumption strengthened by the observed inverse relationship between the mortalities and the wealthy classes.

**Table 4:** Means of transportation to health facility

Means	Pcar/ Truck	Taxi/ Tcy		M-Cycle	PT/ Bus	Bicycle	Boat/ Canoe	Walking	Amb/ Others		No of births
*Age at birth*											
< 20	13.1	23.6		39.5	3.2	1.4	1.3	16.6	0.4		1,337
20–34	21.1	20.6		30.3	4.1	0.8	0.5	22.0	0.3		10,127
35–49	22.7	20.8		27.3	3.2	0.5	0.3	23.5	0.3		1,999
*Birth order*											
1	21.0	23.0		31.1	3.9	0.9	0.7	18.9	0.3		3,519
2–3	20.3	21.8		29.4	4.5	0.8	0.4	22.3	0.3		5,181
4–5	20.0	17.6		30.9	3.2	1.0	0.7	26.5	0.2		2,914
6+	21.1	20.0		34.1	2.9	0.6	0.3	20.0	0.5		1,849
*Antenatal care visits*											
None	11.4	17.0		48.4	1.6	1.2	0.1	18.5	0.2		303
1–3	19.2	21.4		37.4	1.6	1.1	0.5	18.3	0.0		1,058
4+	20.5	21.6		29.4	4.5	0.9	0.6	21.8	0.3		7,388
Missing	12.9	15.3		15.2	2.2	2.1	0.5	51.1	0.0		266
*Residence*											
Urban	24.8	22.9		24.1	4.7	0.6	0.4	22.2	0.2		8,042
Rural	14.1	18.0		40.6	2.7	1.2	0.8	21.0	0.4		5,420
*Zone*											
N-C	13.1	14.2		53.4	2.7	0.1	1.1	15.4	0.1		2,273
N-E	23.6	25.2		32.6	1.8	0.0	1.1	14.1	0.8		1,577
N-W	42.5	31.6		19.9	1.7	1.2	0.0	2.6	0.3		1,955
S-E	13.5	12.6		32.1	2.6	0.2	0.2	36.3	0.4		2,804
S-S	16.2	25.3		31.2	2.3	0.1	1.1	23.6	0.1		1,490
S-W	19.1	22.3		19.5	8.7	2.4	0.4	27.6	0.2		3,364
*Wealth quintile*											
Lowest	8.7	17.9		53.1	1.3	1.2	0.8	16	1.2		879
Second	8.9	16.7		46.3	3	1.5	0.7	21.6	0.4		1,641
Middle	12.5	18		39.5	3.1	0.9	0.9	24.2	0.2		2,836
Fourth	16.4	22.3		30.3	4	0.8	0.3	25.1	0.2		3,701
Highest	35.8	23.8		15.2	5.1	0.5	0.4	18.4	0.2		4,405

Pcar/Truck = Private Car or Truck, Taxi/Tcy = Taxi or Paid Driver or Tricycle, M-Cycle = Motorcycle or Scrooter, PT/Bus = Public Transport or Bus, Boat/Canoe = Boat or Canoe, Amb/Others = Ambulance or Others, N-C = North-central, N-E = North-east, N-W = North-west, S-E = South-east, S-S = South-south, S-W = South-west

Even though transportation by T.P.T and each of the five childhood mortality categories had a statistically insignificant weak association, the positive association implies that as one increases, so does the other. The increased use of this mode of transportation goes hand in hand with an increase in the five childhood mortalities. This contradicts the findings of Tette et al. (2020) that transportation of neonates by taxis proved to be better compared to public bus transportation. However, considering our findings and the submission of Atuoye et al. (2015) that tricycles featured more in the transportation of pregnant women in places where women travel far distances to care facilities, the assumption that places of delivery where this transportation mode was dominant (Fig. 3b) were far from mothers was further strengthened.

The negative association between the five childhood mortality categories and the use of M.S. as maternal transportation (Fig. 3c), though not statistically significant, corroborates the finding of Tette et al. (2020), which reported that there was no significant association between neonates’ mortality and transport mode. From our findings, as one increased, the other decreased, thus suggesting that this mode of transportation might aid in the reduction of childhood mortality because it could be a fast mode of transport in underdeveloped settings. However, the attendant danger of exposing children to strong moving wind could lower their body temperature, thus compromising their health (Weddih et al., 2019; Tette et al., 2020). Coupled with the fact that, except in some rural areas, motorcycles should hardly be used to convey pregnant women for delivery.

The statistically significant negative association between PT.B (Fig. 3d) and each of the identified childhood mortalities contradicts the findings of Tette et al. (2020) that children transported by public bus had poorer outcomes. This might not be unconnected with the situation where PT.B is one of the most affordable means of transportation for most residents of Low- or Middle-Income Countries (LMICs). Thus, a more efficient and affordable public bus transportation system will aid reduction in mortality in the southwestern part of the country, where the use of PT.B as maternal transport is concentrated.

As would be expected, transportation by Bicycle (Fig. 3e) and each of the five childhood mortality categories had positive associations (Fiagbe et al., 2012; Atuoye et al., 2015), though not statistically significant, suggesting that this means of transportation could contributes to increased childhood mortality. Apart from the inconvenience associated with this means of maternal transportation, the danger to the mother and child is enormous. The positive associations, though not statistically significant, suggested that the transport mode can lead to more deaths and should be discouraged as a means of maternal transportation.

The use of a B.C (Fig. 3f) as means of maternal transportation and each of the identified categories of childhood mortality had a non-significant negative associations, with the implication that the means of transportation can contribute to the reduction in childhood mortality. This might be adduced to the use of high-speed overboard engines that ensured rapid movement. Hence, the use of this means of maternal transport needs to be encouraged in riverine and coastal communities as it has the potential to aid in the quick transportation of children to care facilities, thus lowering childhood mortality.

The five childhood mortalities and walking (Fig. 3g) to the healthcare facility of delivery had statistically significant positive associations, implying that trekking to delivery facilities could reduce childhood mortality, although it is discouraged (Thaddeus & Maine, 1994; Fiagbe et al., 2012) as a means of maternal transportation. The probable deduction is that most delivery facilities were within walking distances of the women. This is clarified in Table 2; Fig. 3g: the southern part of the country had lower cases of childhood mortalities and the highest percentages of women who trekked to where they delivered their newborns. Therefore, as easy as it was for them to trek to deliver, it will be equally easy to take children for medical care, thus preventing mortality.

Transportation by A.O had a positive association with all childhood mortality categories, although not statistically significant, contrary to the findings of Singh et al. (2016). The mode was predominantly used in the northeastern part of the country (Fig. 3h). The weak positive association implies that the means of transportation should be discouraged, but should ambulance services be discouraged? The most probable suggestion is that other means of maternal transportation in the category, apart from an ambulance, should be discouraged. Increased use of ambulances in the region might have resulted from the increased presence of foreign aid agencies helping to address the humanitarian crisis caused by insurgent activity in the region.

Earlier studies have shown that, among other things, long distances to the delivery facilities and underdeveloped public transportation directly affect childhood mortality (Banke-Thomas, 2021; Ijdi et al., 2022). This has been further corroborated by the findings of this study. A well organized and efficient public transport system will go a long way in aiding less previledged women and children to promptly access healthcare, thus reducing both maternal- and childhood mortalities in Nigeria.

The potential of maternal transport modes to reduce childhood mortality has been demonstrated in this study. Private cars or trucks may help reduce childhood mortality, but in places where health facilities are not far from mothers. There is a need to organize public modes of transportation to be more efficient so that women are encouraged to use such modes for maternal and child health care services. Steps should be taken to reduce the travel distances to health facilities to walking distances for mothers. Moreover, arrangements should be made to make emergency maternal transportation readily available.

## Data Availability

The data underlying this study is in the public domain.
